# Dietary menthol-induced TRPM8 activation enhances WAT “browning” and ameliorates diet-induced obesity

**DOI:** 10.18632/oncotarget.20540

**Published:** 2017-08-24

**Authors:** Changyu Jiang, Mingzhu Zhai, Dong Yan, Da Li, Chen Li, Yonghong Zhang, Lizu Xiao, Donglin Xiong, Qiwen Deng, Wuping Sun

**Affiliations:** ^1^ Department of Pain Medicine and Shenzhen Municipal Key Laboratory for Pain Medicine, The Affiliated Nanshan People’s Hospital of Shenzhen University, Shenzhen Municipal Sixth People’s Hospital, Shenzhen 518060, China; ^2^ Institute of Science and Technology Austria, Am Campus 1, Klosterneuburg 3400, Austria; ^3^ Center of Reproductive Medicine, Department of Obstetrics and Gynecology, Shengjing Hospital of China Medical University, Shenyang 110004, China; ^4^ Laboratory of Medicinal Plant, School of Basic Medicine, Laboratory of Chinese Herbal Pharmacology, Oncology Center, Renmin Hospital and Hubei Key Laboratory of Wudang Local Chinese Medicine Research, Hubei University of Medicine, Hubei 442000, China; ^5^ Department of Infectious Diseases and Shenzhen Municipal Key Laboratory for Endogenous Infection, The Affiliated Nanshan People’s Hospital of Shenzhen University, Shenzhen Municipal Sixth People’s Hospital, Shenzhen 518060, China

**Keywords:** WAT, beige adipocytes, thermogenesis, browning, obesity

## Abstract

Beige adipocytes are a new type of recruitable brownish adipocytes, with highly mitochondrial membrane uncoupling protein 1 expression and thermogenesis. Beige adipocytes were found among white adipocytes, especially in subcutaneous white adipose tissue (sWAT). Therefore, beige adipocytes may be involved in the regulation of energy metabolism and fat deposition. Transient receptor potential melastatin 8 (TRPM8), a Ca^2+^-permeable non-selective cation channel, plays vital roles in the regulation of various cellular functions. It has been reported that TRPM8 activation enhanced the thermogenic function of brown adiposytes. However, the involvement of TRPM8 in the thermogenic function of WAT remains unexplored. Our data revealed that TRPM8 was expressed in mouse white adipocytes at mRNA, protein and functional levels. The mRNA expression of *Trpm8* was significantly increased in the differentiated white adipocytes than pre-adipocytes. Moreover, activation of TRPM8 by menthol enhanced the expression of thermogenic genes in cultured white aidpocytes. And menthol-induced increases of the thermogenic genes in white adipocytes was inhibited by either KT5720 (a protein kinase A inhibitor) or BAPTA-AM. In addition, high fat diet (HFD)-induced obesity in mice was significantly recovered by co-treatment with menthol. Dietary menthol enhanced WAT “browning” and improved glucose metabolism in HFD-induced obesity mice as well. Therefore, we concluded that TRPM8 might be involved in WAT “browning” by increasing the expression levels of genes related to thermogenesis and energy metabolism. And dietary menthol could be a novel approach for combating human obesity and related metabolic diseases.

## INTRODUCTION

Obesity is a serious health problem that is implicated in various diseases including type II diabetes, hypertension, coronary heart diseases and cancer [1], and it is characterized by increased adipose tissue mass that results from increased fat cell size and number, suggesting that the main contributor to obesity is adipose tissue [2]. There are two types of adipose tissues, white adipose tissue (WAT) and brown adipose tissue (BAT). The functions of these two types of adipose tissues are almost the opposite of one another [3]. The main function of BAT is energy dissipation, or generating heat, and BAT is a major site for mammalian non-shivering thermogenesis with mitochondrial uncoupling protein 1 (UCP1) [4]. When activated, UCP1 uncouples the respiratory chain and heat is generated [5]. It has also been reported that a new type of recruitable brownish adipocytes, termed “beige adipocytes”, was found among white adipocytes, especially in subcutaneous WAT (sWAT) [6]. Beige adipocytes are recruited especially after a short-term cold challenge or giving a β3-adrenergic receptor agonist treatment, which are very similar to brown adipocytes, with highly mitochondrial membrane uncoupling protein 1 (UCP1) expression and thermogenesis [7, 8]. This novel finding highlights the crucial role for beige cells in the regulation of energy metabolism and fat deposition. Thus, enhancing of beige adipocytes activity in WAT could be a promising target for the prevention and therapy of human obesity, and understanding the molecular mechanisms for thermogenesis in “beige adipocytes” is the subject of intense investigation.

The concentration of free intracellular Ca^2+^ ([Ca^2+^]_i_) and the amplitude of its fluctuations have primary importance for survival and function in a plethora of cell types [9]. For many cells there have been extensive studies of [Ca^2+^]_i_ signals, including investigation of the plasma membrane ion channels that directly permit Ca^2+^ influx or control Ca^2+^ influx indirectly. There is, by contrast, relatively little known about Ca^2+^ signaling in adipose tissue, despite its suggested importance [10].

A major class of Ca^2+^-permeable channels is constituted by the transient receptor potential (TRP) ion channels, most of which are non-selective Ca^2+^-permeable cation channels [11]. TRP channels have six transmembrane (TM) domains (TM1 to TM6) and a pore loop between TM5 and TM6 with both N- and C-termini in the cytosol [12]. The TRP channel superfamily is now classified into six subfamilies in mammals: TRPV (Vanilloid), TRPC (Canonical), TRPM (Melastatin), TRPML (Mucolipin), TRPP (Polycystin) and TRPA (Ankyrin). TRP channels are unique cellular sensors characterized by promiscuous activation mechanisms, including thermal and mechanical activation [13]. The TRP superfamily of ion channels is widely expressed and has multiple functions [14–16]. The main signaling pathway which TRP channels involved are derived TRP channels activation-induced calcium influx and triggered [Ca^2+^]_i_. Among the members of TRP channel family, TRPM8 is initially named TRPP8 for its homology with TRP family members and cloned by Mckemy D et al. in 2002 [17]. TRPM8 has been reported to be expressed in a subset of sensory neurons, where it acts as a direct sensor of cold stimuli (temperature threshold lower than 30°C) and cooling agents such as menthol or icillin [17–21].

Several TRP channels are reported to be involved in adipocyte biology, data have shown that activation of either TRPV1 [22] or TRPV3 [23] prevented adipogenesis in 3T3-L1 pre-adipocytes and played an anti-adipogenic role *in vivo*. It has also been reported that TRPV2 is functionally expressed in brown adipocytes and is involved in BAT thermogenesis and differentiation [24–26]. TRPV4 negatively regulates mitochondrial oxidation, knockdown of TRPV4 facilitated UCP1 expression in adipocyte cell line and TRPV4KO mice exhibited less obese when fed with high fat diet (HFD) [27]. Moreover, it has already reported that TRPM8 stimulation by its ligands increased UCP1 expression in brown adipocytes and BAT through PKA phosphorylation [28]. And activation of TRPV1 by capsaicin or TRPM8 by cold temperature or menthol enhanced UCP1 expression [29, 30]. However, there is still no detailed understanding of menthol-induced activation of TRPM8 in WAT “browning”.

In this study, we demonstrated that TRPM8 was functionally expressed in mouse white adipocytes in culture. We also found that menthol-induced activation of TRPM8 increased thermogenic gene expression in both cultured white adipocytes and sWAT. Moreover, dietary menthol enhanced WAT “browning” and significantly suppressed HFD-induced obesity. In addition, dietary menthol reduced insulin resistance in HFD-induced obesity mice as well. These data suggested that TRPM8 might be involved in WAT “browning” by increasing the expression levels of genes related to thermogenesis and energy metabolism. Dietary menthol could be a promising approach for the treatment and prevention of human obesity and related metabolic diseases.

## RESULTS

### TRPM8 is functionally expressed in the differentiated mouse white adipocytes in culture

**Figure 1:**
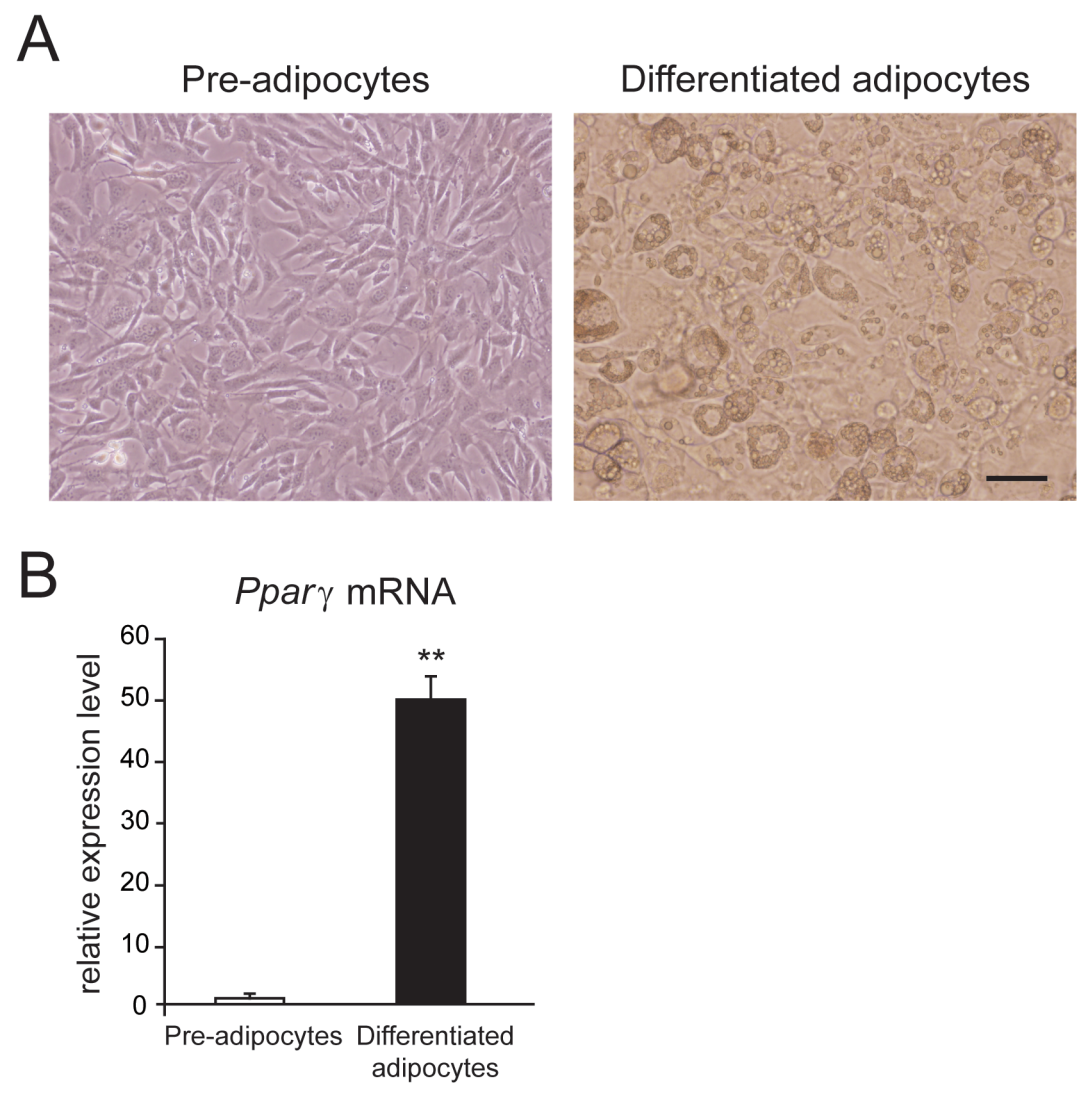
Mouse white adipocytes in culture **(A)** Phase-contrast images of primary cultured mouse white pre-adipocytes and differentiated adipocytes. **(B)** mRNA expression level of *peroxisome proliferator-activated receptor γ* (*Pparγ*) in mouse pre-adipocytes and differentiated adipocytes. Data are presented as mean ± SEM, n = 6; ** *P* < 0.01 *vs.* pre-adipocytes. Unpaired Student’s *t*-test.

First of all, we established the primary culture system of mouse white adipocytes. Figure 1A shows the images of primary cultured mouse white pre-adipocytes and differentiated adipocytes. To confirm the differentiation of mouse white aidpocytes, we examined the mRNA expression of *peroxisome proliferator-activated receptor γ* (*Pparγ*), which is a key transcriptional factor for adipogenesis. We observed that the mRNA expression of *Pparγ* was significantly increased in the differentiated white adipocytes than in pre-adipocytes from mouse sWAT (Figure 1B). These results suggested that the primary culture for mouse white adipocytes was succeeded.

**Figure 2:**
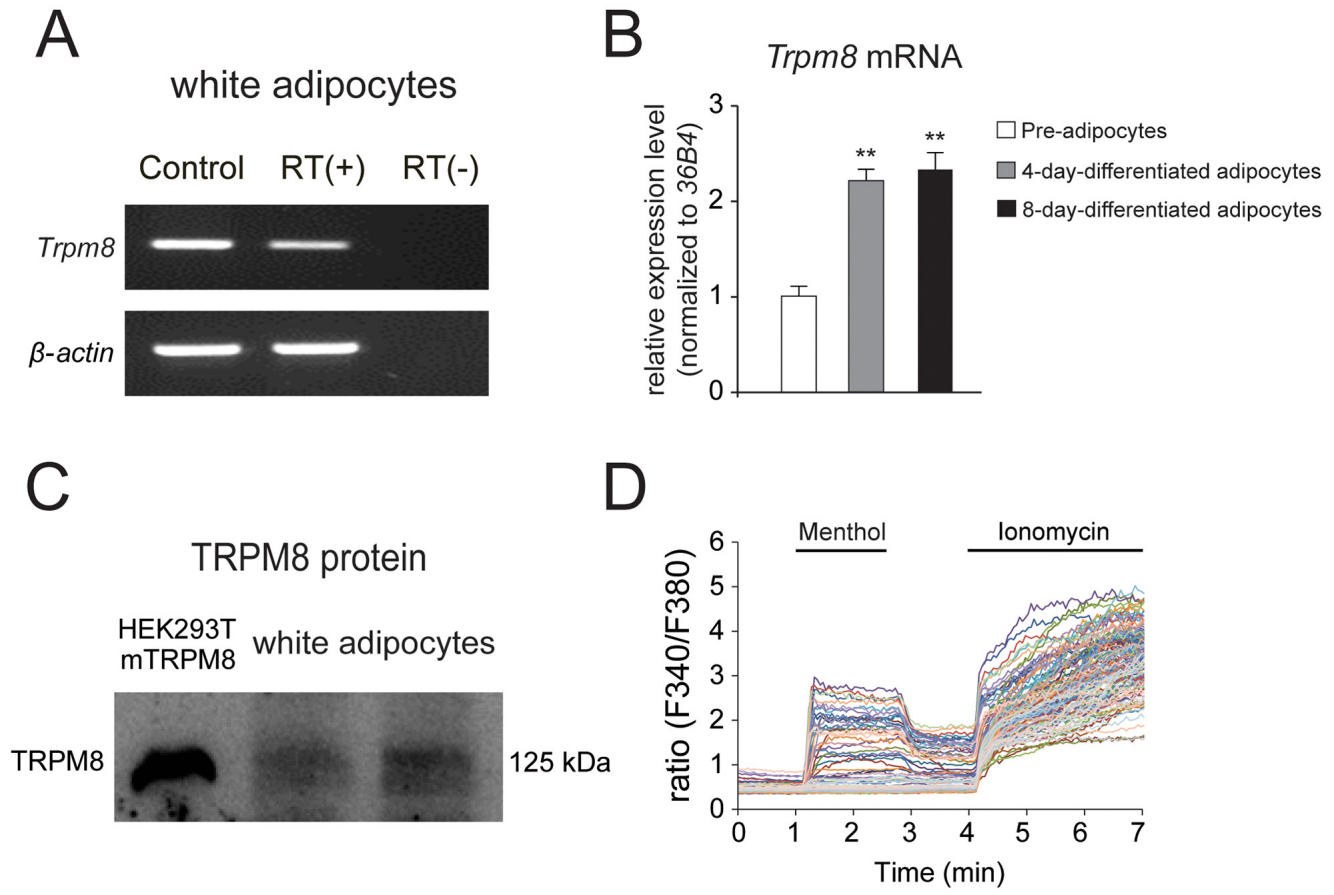
TRPM8 is functionally expressed in mouse differentiated white adipocytes **(A)** RT-PCR analysis of the expression of *Trpm8* and *β-actin* using mouse differentiated white adipocytes with (RT (+)) and without (RT (-)) reverse transcription (RT). Control (Ct.) lanes indicate the results with each plasmid DNA as a template. **(B)** Results of real-time RT-PCR analysis of *Trpm8* expression using mouse pre-adipocytes, 4-day-differentiated adipocytes and 8-day-differentiated adipocytes. mRNA expression levels were normalized to that of the ribosomal protein gene (*36B4)*, a housekeeping gene un-affected by adipogenesis. Data are presented as mean ± SEM, n = 6. **(C)** Western blot result of TRPM8 protein in mouse differentiated white adipocytes. **(D)** Changes in intracellular Ca^2+^ concentration ([Ca^2+^]_i_) in mouse white adipocytes response to a TRPM8 agonist, 500 μM menthol. Five μM ionomycin was used to confirm cell viability.

To examine the expression of TRPM8 in mouse white adipocytes, we first examined TRPM8 expression in cultured white adipocytes. RT-PCR and real-time RT-PCR analyses revealed that *Trpm8* (Figure 2A and 2B), but not *Trpa1* mRNA (Supplementary Figure 1A), was expressed in cultured mouse white adipocytes. Moreover, the mRNA level of *Trpm8* was significantly increased in both 4-day-differentiated and 8-day-differentiated white adipocytes than in pre-adipocytes (Figure 2B). We further examined the protein expression of TRPM8 in the differentiated adipocytes by Western blotting. And TRPM8 protein bands were observed in the differentiated white adipocytes lanes (Figure 2C). Next, we examined the functional expression of TRPM8 in mouse white adipocytes using a Ca^2+^-imaging method. A TRPM8 agonist, menthol increased [Ca^2+^]_i_ (Figure 2D), indicating that TRPM8 is functional expressed in white adipocytes in culture. Adipocytes showed increases in [Ca^2+^]_i_ upon ionomycin application, indicating the viability of the differentiated adipocytes. These results revealed that TRPM8 was functionally expressed in the differentiated mouse white adipocytes.

### Menthol-induced TRPM8 activation increases thermogenic gene expression in cultured mouse white adipocytes

**Figure 3:**
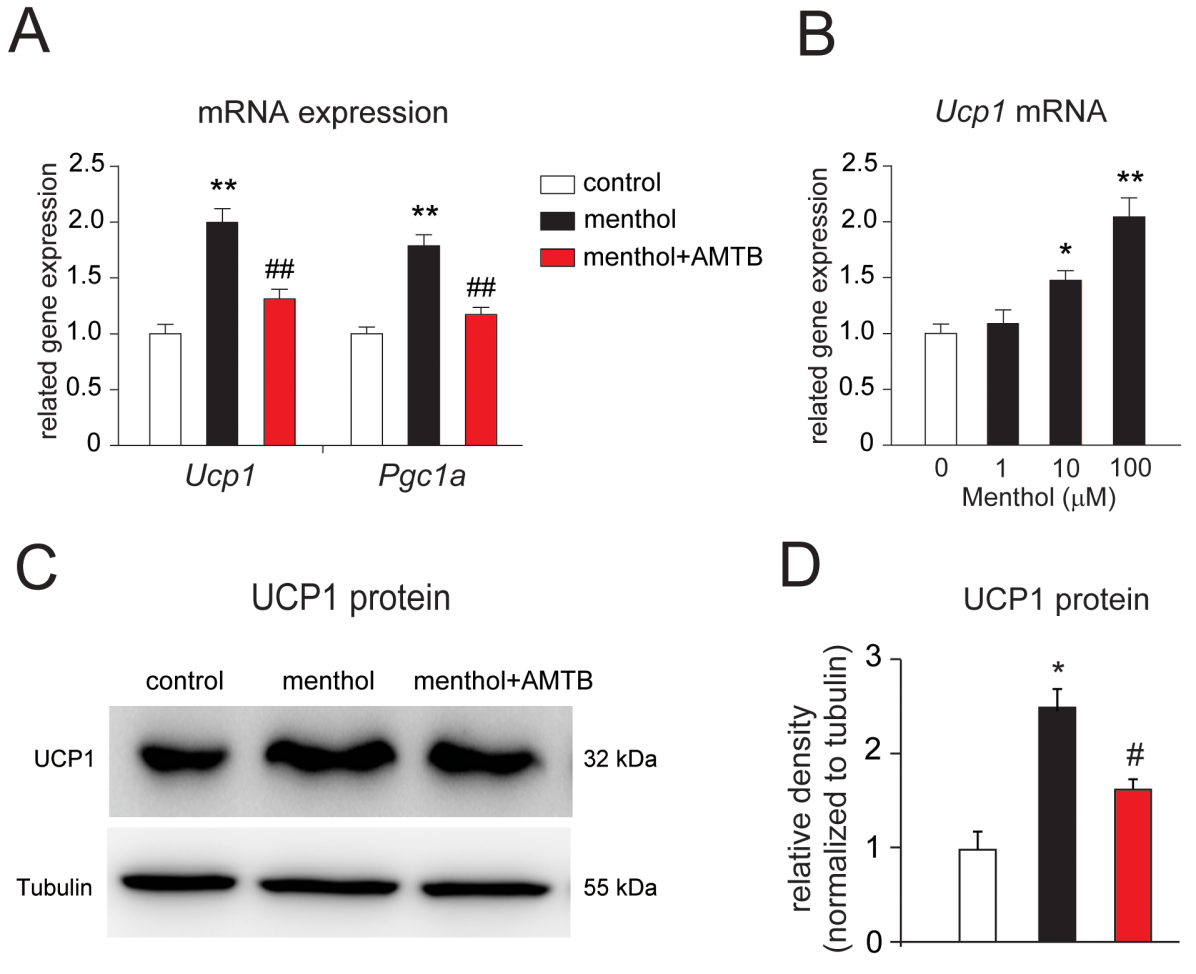
Menthol enhances thermogenic gene expression in mouse white adipocytes in culture **(A)** Changes in *Uncoupling protein 1* (*Ucp1*) and *peroxisome proliferator-activated receptor gamma coactivator 1-a* (*Pgc1a*) mRNA expression in the differentiated white adipocytes treated with menthol alone or menthol (100 μM) plus a TRPM8 blocker, *N*-(3-Aminopropyl)-2-[(3-methylphenyl)methoxy]-*N*-(2-thienylmethyl)benzamide hydrochloride salt (AMTB, 10 μM) for 4 h. Data are presented as mean ± SEM, n = 6; ** *P* < 0.01 *vs.* control; ## *P* < 0.01 *vs.* menthol group. One-way ANOVA followed by 2-tailed *t*-test with Bonferroni correction. **(B)** Effects of different concentrations of menthol on the mRNA expression of *Ucp1* in mouse white adipocytes in culture after incubation for 4 h. Data are presented as mean ± SEM, n = 6; * *P* < 0.05, ** *P* < 0.01 *vs.* control. One-way ANOVA followed by 2-tailed *t*-test with Bonferroni correction. **(C)** Western blot result of UCP1 and tubulin from cultured mouse white adipocytes treated with menthol alone or menthol (100 μM) plus AMTB (10 μM) for 1 day. **(D)** Comparison of UCP1 protein levels in cultured mouse white adipocytes treated with menthol alone or menthol plus AMTB. Mean ± SEM, n = 6; ** *P* < 0.05 *vs.* control; # *P* < 0.05 *vs.* menthol group. One-way ANOVA followed by 2-tailed *t*-test with Bonferroni correction.

To investigate the effects of menthol on the differentiated white adipocytes, we examined the thermogenic gene, *Ucp1* and *peroxisome proliferator-activated receptor gamma coactivator 1-a* (*Pgc1a*) mRNA expression in white adipocytes incubated in culture for 8 h with menthol or menthol plus a specific TRPM8 blocker, *N*-(3-Aminopropyl)-2-[(3-methylphenyl) methoxy]-*N*-(2-thienylmethyl)benzamide hydrochloride salt (AMTB) [31]. We observed that the gene expression of both *Ucp1* and *Pgc1a* mRNA were significantly increased in mouse white adipocytes in culture treated with menthol alone, and recovered when incubated with menthol plus AMTB for 8 h (Figure 3A). Moreover, menthol-induced increases of *Ucp1* mRNA expression level in adipocytes exhibited a dose-dependent manner (Figure 3B). Next, we examined UCP1 protein expression level in cultured white adipocytes treated with menthol alone or menthol plus AMTB. We found that UCP1 protein band was denser in menthol treated adipocytes lane than control lane and menthol plus AMTB treated adipocytes lane (Figure 3C). Quantitative analysis result of UCP1 protein level was significantly increased in menthol treated adipocytes, and recovered in menthol plus AMTB-treated adipocytes (Figure 3D). These results suggested that menthol-induced increases in UCP1 and PGC1a gene expression occurred via TRPM8 activation in mouse white adipocytes in culture. We also asked whether TRPM8 is involved in adipogenesis of white adipocytes. Continuous treatment with menthol (either 30 μM or 100 μM) during the whole 8 days adipogenesis, did not affect oil red O signals (Supplementary Figure 2A). And the number of differentiated adipocytes and triglyceride levels were also not changed upon continuous treatment with menthol during the whole process of adipogenesis (Supplementary Figure 2B and 2C). These results demonstrated that activation of TRPM8 did not affect adipogenesis of white adipocytes.

### Menthol-induced thermogenic gene expression increases are through [Ca^2+^]_i_ increase-induced PKA phosphorylation

**Figure 4:**
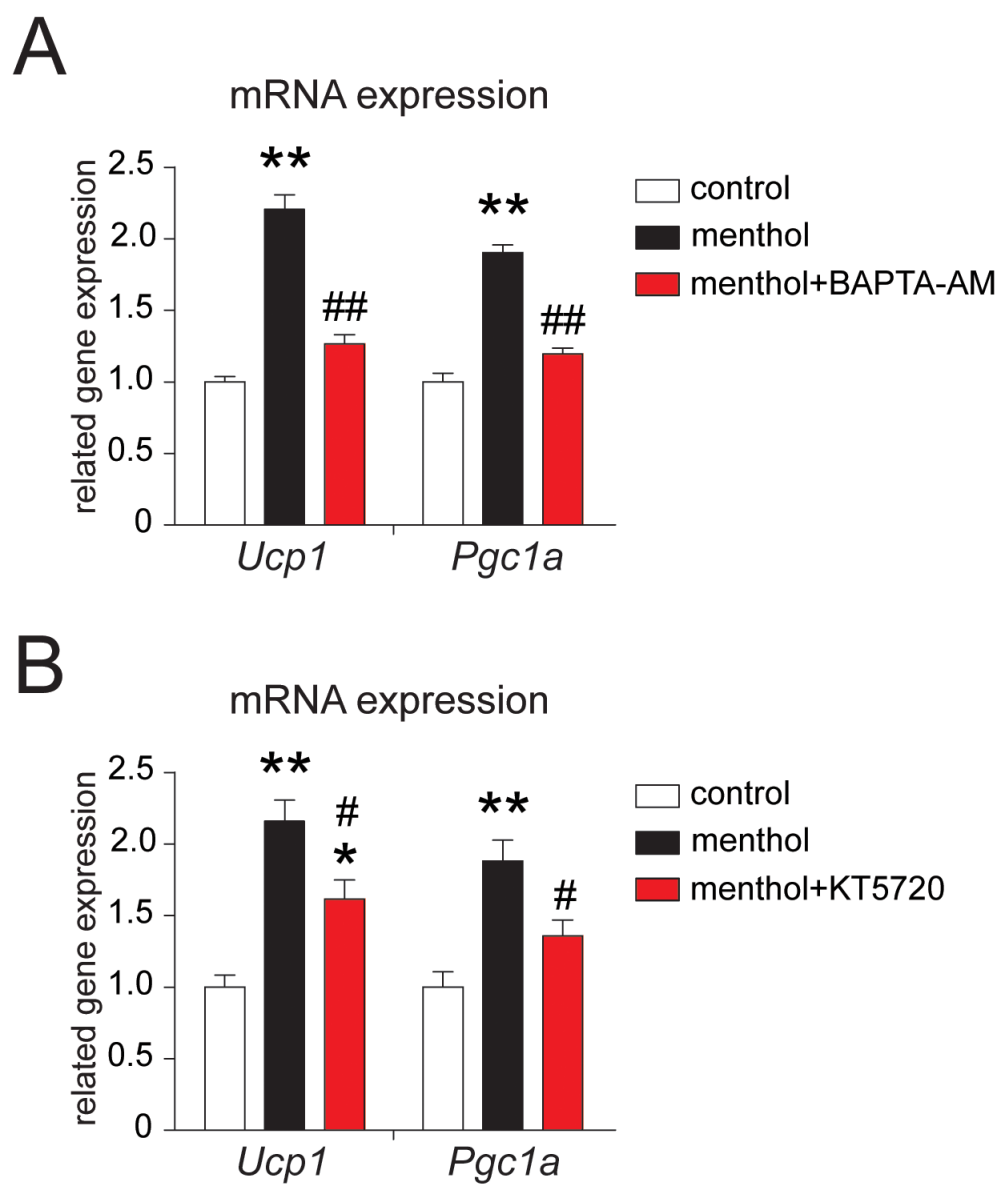
Effects of BAPTA-AM or KT5720 on menthol-induced thermogenic program in white adipocytes from mouse **(A)** mRNA expression of *Ucp1* and *Pgc1a* in cultured mouse white adipocytes treated with menthol (100 μM) alone or menthol (100 μM) plus BAPTA-AM (10 μM) for 4 h. BAPTA-AM is a membrane-permeable calcium chelator. **(B)** mRNA expression of *Ucp1* and *Pgc1a* in cultured mouse white adipocytes treated with menthol (100 μM) alone or menthol (100 μM) plus KT5720 (1 μM). KT5720 is a specific PKA inhibitor. Mean ± SEM, n = 6; ** *P* < 0.05 *vs.* control; # *P* < 0.05 *vs.* menthol group. One-way ANOVA followed by 2-tailed *t*-test with Bonferroni correction.

To explore the mechanism of TRPM8 activation-induced thermogenic program in white adipocytes, we examined the effect of BAPTA-AM, a membrane permeable calcium chelator, and KT5720, a specific PKA inhibitor on thermogenic gene expression in white adipocytes. We found that menthol-induced thermogenic gene expression increases in mouse white adipocytes were significantly inhibited by co-application with either BAPTA-AM (Figure 4A) or KT5720 (Figure 4B). These results suggested that menthol-induced [Ca^2+^]_i_ increases enhanced *Ucp1* and *Pgc1a* mRNA expression may via PKA phosphorylation in white adipocytes.

### Menthol enhances WAT “browning” and suppresses HFD-induced obesity in mice

**Figure 5:**
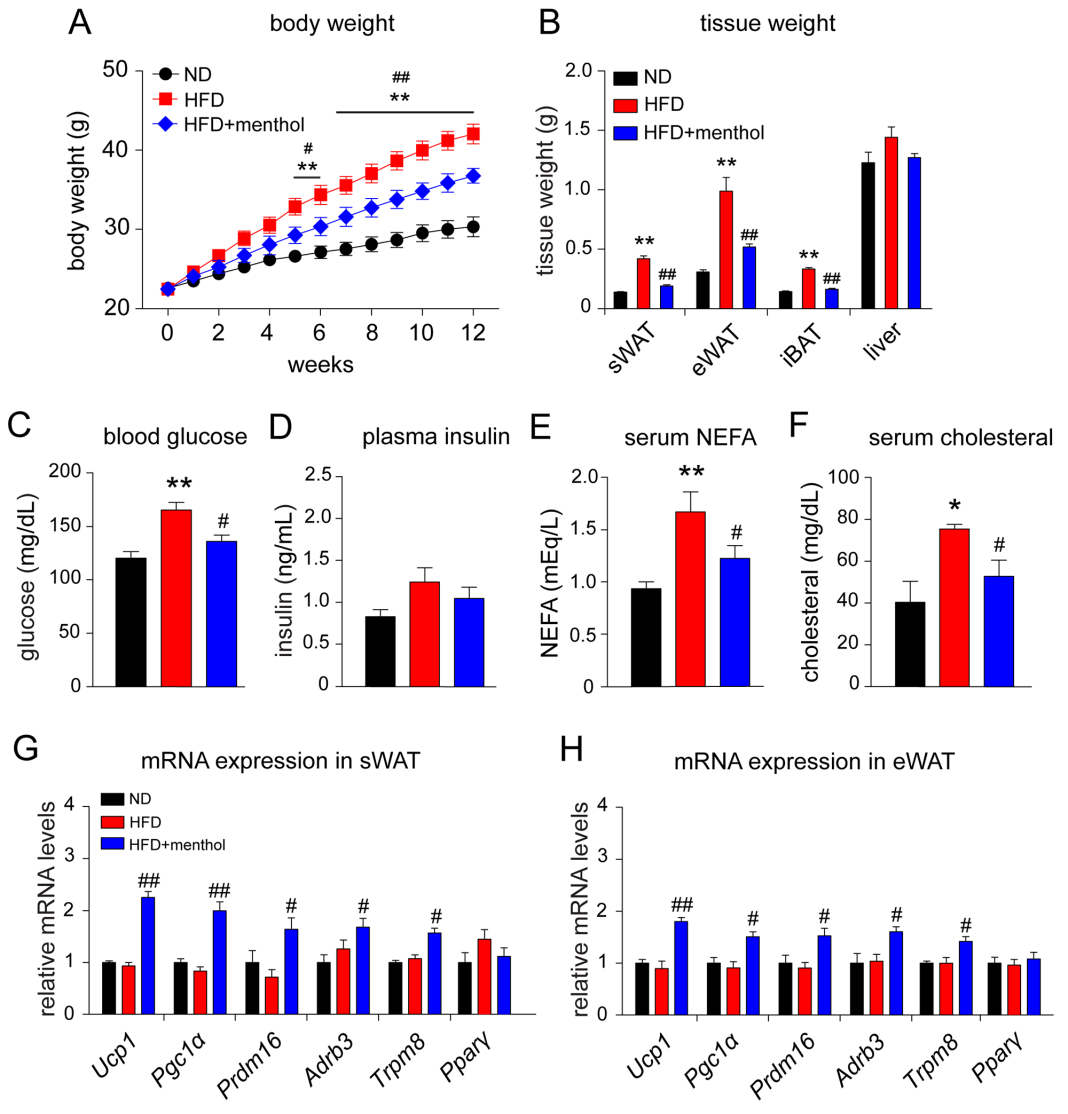
Effects of dietary menthol on high fat diet-induced obesity mice **(A)** Body weight changes on high fat diet (HFD)-induced obesity mice treated with or without menthol for 12 weeks continuously from 6 weeks of age. **(B)** Weights of tissues related to energy metabolism in mice after 1-week HFD treatment alone or with menthol (1% in HFD diet). **(C** and **D)** Blood glucose level (C) and plasma insulin level (D) in mice after 12-week HFD treatment alone or with menthol. **(E** and **F)** Serum non-sterified free fatty acid (NEFA) (E) and serum cholesterol level (F) in mice after 12-week HFD treatment alone or with menthol. **(G** and **H)** Quantitative RT-PCR results of thermogenic genes and *Trpm8* in subcutaneous WAT (sWAT) and epididymal WAT (eWAT) from mice treated with HFD alone or HFD plus menthol. All data are presented as mean ± SEM, n = 8; *P < 0.05; **P < 0.01 *vs.* ND group. # *P* < 0.05; ##*P* < 0.01 *vs.* HFD group. One-way ANOVA followed by 2-tailed *t*-test with Bonferroni correction.

We next asked whether menthol could enhance thermogenic gene expression *in vivo* and reduce HFD-induced obesity. Our results demonstrated that dietary menthol treatment for 18 weeks significantly reduced HFD-induced body weight gain in mice (Figure 5A). Menthol significantly reduced tissue weights of sWAT, epididymal WAT (eWAT) and interscapular BAT (iBAT) as well, compared with HFD treatment alone (Figure 5B). Moreover, menthol reduced HFD-induced hyperplasia, but no significant effect on plasma insulin level (Figure 5C and 5D). Serum NEFA and cholesterol levels were also significantly reduced in HFD plus menthol treatment group, compared with HFD treatment alone (Figure 5E and 5F). These results suggested that menthol effectively reduced HFD-induced obesity in mice. In addition, mRNA expression levels of *Ucp1*, *Pgc1a*, *PR domain containing 16* (*Prdm16*), *beta-3 adrenergic receptor* (*Adrb3*) and *Trpm8* were significantly increased upon HFD plus menthol treatment in both sWAT and eWAT, whereas *Pparγ* expression level was not changed (Figure 5G and 5H). And the mRNA expression levels of *Ucp1*, *Pgc1a*, *Prdm16*, *Adrb3*, *Trpm8* and *Pparγ* were not changed between ND and HFD group. These results suggested that menthol increased thermogenic program in WAT and reduced HFD-induced obesity in mice.

### Dietary menthol enhances “browning” and reduces adipocyte size in sWAT of mice treated with HFD

**Figure 6:**
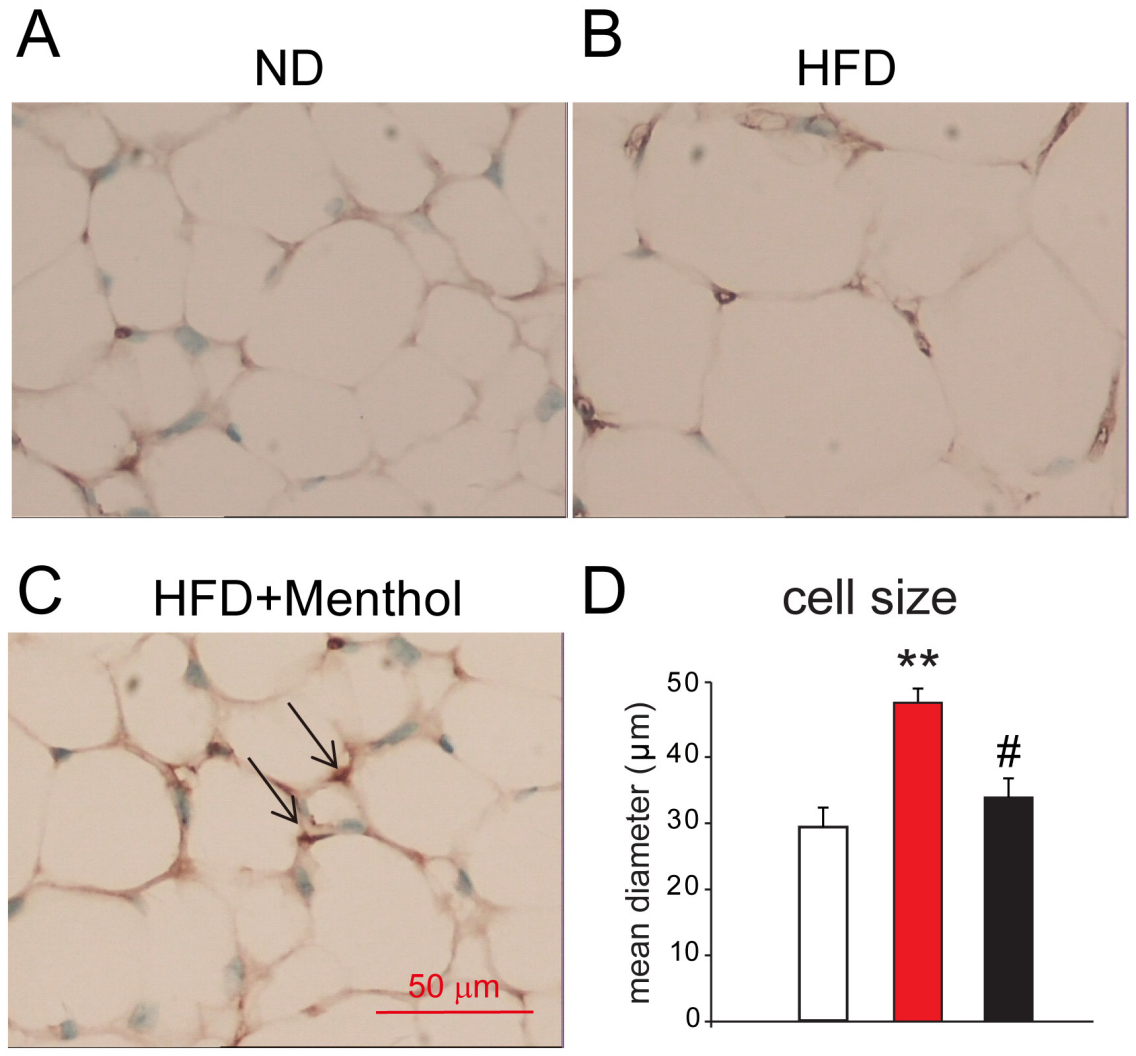
Immunohistochemistry results of UCP1 staining in sWAT among mice treated with ND, HFD or HFD+menthol **(A, B** and **C)** Representative images from immunohistochemistry for UCP1 (brown stain) in sWAT from mice treated with ND (A), HFD (B) and HFD+menthol (C). UCP1-expressing cells are indicated by arrows. **(D)** Mean diameters of adipocytes of sWAT from mice treated with ND, HFD and HFD+menthol. Mean ± SEM, n = 5; **P < 0.01 *vs.* WT group; #*P* < 0.05 *vs.* HFD group. One-way ANOVA followed by 2-tailed *t*-test with Bonferroni correction.

To further examine the effect of dietary menthol on obese mice, we performed immunohistochemistry experiment and found that UCP1 positive adipocytes (beige adipocytes) were increased in sWAT from mice treated with HFD plus menthol, compared with ND treatment or HFD treatment (Figure 6A-6C). Moreover, adipocyte mean diameter was also significantly reduced in sWAT from mice treated with HFD plus menthol (Figure 6D), compared with ND treatment or HFD treatment. These results further suggested that dietary menthol reduced HFD-induced obesity by enhancing sWAT “browning” (beige adipocytes recruitment) in mice.

### Dietary menthol improves glucose metabolism *in vivo*

**Figure 7:**
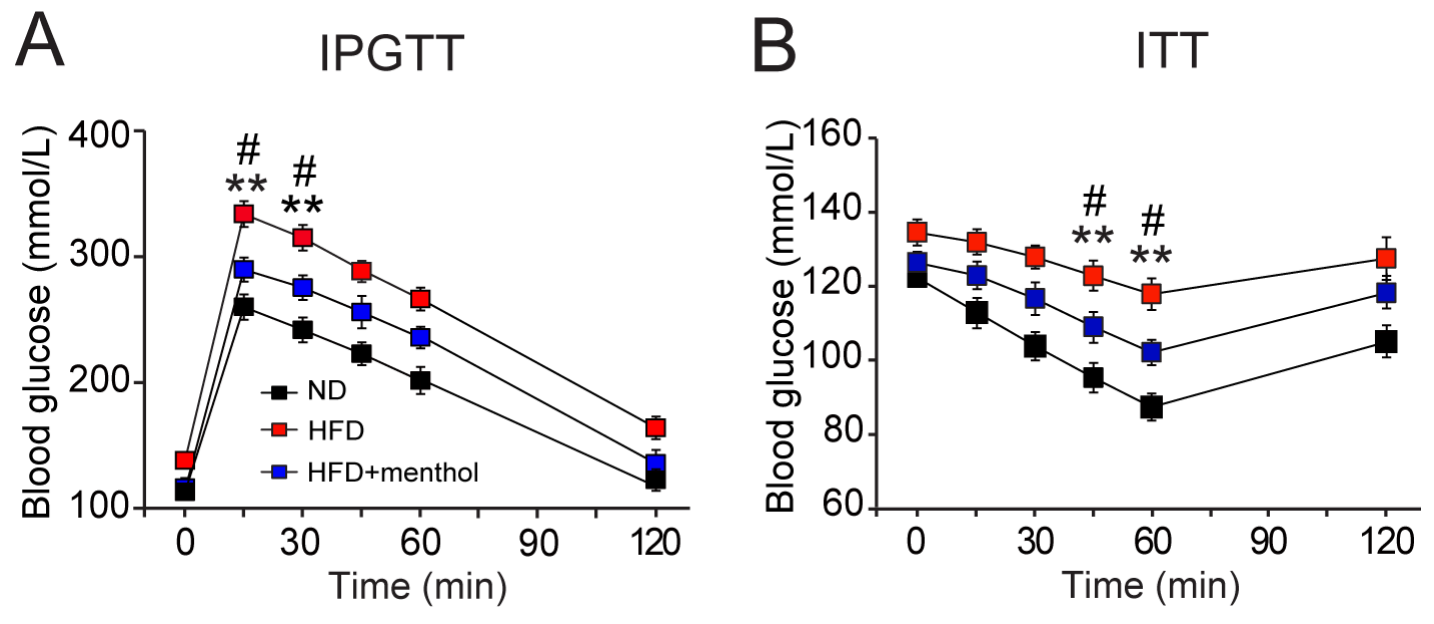
Effects of dietary menthol on glucose metabolism in HFD-induced obesity mice **(A)** Plasma glucose level changes in mice treated with HFD alone or HFD plus menthol (1% in HFD) after glucose loading. **(B)** Plasma glucose levels changes in mice treated with HFD alone or HFD plus menthol after insulin administration. IPGTT indicates intraperitoneal glucose tolerance tests and ITT indicates insulin tolerance tests. Data indicates mean ± SEM, n = 8; ** *P* < 0.01 *vs.* ND group. # *P* < 0.05 *vs.* HFD+menthol group. Repeated measures ANOVA followed by Dunnett’s pot hoc test were performed, statistical differences by Dunnett’s post hot test were shown.

Because menthol diet effectively reduced the phenotype of HFD-induced obesity in mice, we next examined the glucose metabolism in mice after treatment with HFD plus menthol or HFD alone by IPGTT and ITT experiments. Blood glucose levels in 15 and 30 min after glucose administration (i.p. 2 g/kg body weight) were significantly decreased in HFD plus menthol treatment group, compared with HFD group (Figure 7A). Moreover, blood glucose levels in 45 and 60 min after insulin injection (i.p. 1 unit/kg body weight) were also significantly decreased in HFD plus menthol treatment group, compared with HFD group (Figure 7B). Our data revealed that dietary menthol significantly improved IPGTT and ITT after HFD treatment in mice. These results further suggested that dietary menthol improved glucose metabolism in HFD-induced mice.

## DISCUSSION

Adipose tissue plays key roles in systemic energy homeostasis and metabolic regulation [27], and adipose tissue is a major contributor to obesity [2]. WAT is the primary depot for energy storage in mammals [32] and BAT is an important component in whole-body energy homeostasis through the dissipation of stored chemical energy in the form of heat [33]. Recently, a new type of recruitable brownish adipocyte, termed “beige adipocytes” (also known as “bright adipocytes”), among white adipocyte, especially in sWAT has been reported to be a promising target [6]. Beige adipocytes are recruited after a short-term cold challenge or treatment with β3-adrenergic receptor agonists. TRPM8 has been reported to be functionally expressed in brown adipocytes, and activation of TRPM8 by menthol up-regulated the expression level of UCP1 [28]. In this study, we provided several lines of evidence that TRPM8, the most important cold sensing receptor in mammals is expressed in sWAT. And menthol-induced TRPM8 activation resulted in [Ca^2+^]_i_ increases, which further led to sWAT “browning” and reduced body weight gain and insulin resistance in HFD-induced obesity mice. Therefore, enhancement of TRPM8 activities by dietary food, such as menthol, could pave an intriguing way for the treatment and prevention of human obesity and related metabolic diseases.

Activation of TRPM8 by dietary menthol increased the expression of thermogenic gene, UCP1 and PGC1α in sWAT, in another words, sWAT “browning”, which mimic the effects induced by cold exposure in stimulating these cells to behave as brownish adipocytes or by recruiting beige adipocytes as recently reported [6]. It has also been reported that menthol treatment or cold exposure caused a TRPM8-dependent increase in core body temperature, which might be related to an increase in UCP1 expression [34]. Moreover, intragastric administration of menthol enhanced BAT thermogenesis in mice [35, 36]. Although it was reported that menthol activates TRPA1 as well [37], TRPM8 activation is mainly involved in the effect of dietary menthol because *Trpa1* mRNA was not detected in sWAT of mice (Supplementary Figure 1). In addition, TRPM8 was demonstrated to be expressed in a human adipocyte cell line in which TRPM8 activation induced UCP1 expression, mitochondrial activation and heat production [30]. The mRNA expression level of *Trpm8* is significantly increased during the differentiation of adipocytes, suggesting the significance of TRPM8 in adipocyte thermogenesis. This study suggested that activation of TRPM8 enhances BAT thermogenesis, which could offer promising approaches to treat and prevent obesity.

Menthol has no effect in the differentiation of white adipocytes when continuously treat pre-adipocytes with different concentrations of menthol during the whole process of adipocyte differentiation (Supplementary Figure 2). These data could also explain the reason of the low expression of *Trpm8* mRNA in pre-adipocytes. In coincide with our data, it has been reported that menthol has no effects on the expression levels of PPARγ or bone morphogenetic protein 7, both of which are involved in the induction of the differentiation of brown adipocytes [28]. In the “browning” effects of cold exposure on white adipocytes is not associated with an increase of PDRM16 gene expression despite an increase in UCP1 expression [38]. UCP1 is also a key contributor in the regulation of diet-induced thermogenesis [39]. Several studies have reported that UCP1 activity and expression in BAT can be activated by p38/MAPK, AMPK, PI3K, and cAMP-dependent PKA [40, 41]. In addition, ectopic expression of PGC1α in WAT induces the expression of UCP1 [41]. Our findings revealed that TRPM8 activation-mediated [Ca^2+^]_i_ increases enhanced sWAT thermogenesis may through PKA phosphorylation because menthol-induced increases of the the mRNA expression levels of *Ucp1* and *Pgc1α* were significantly inhibited by either KT5720 (protein kinase A inhibitor) or BAPTA-AM (a membrane-permeable calcium chelator) in cultured white adipocytes. It was reported that PKA phosphorylates the transcription factor cAMP-response element binding protein and PGC1α, which further activates the expression of UCP1 [42]. Moreover, It was reported that TRPM8 in HEK293 cells can be regulated by the Gi protein/adenylate cyclase/cAMP/PKA signaling cascade [43, 44]. These findings suggested that dietary menthol-induced TRPM8 activation increases [Ca^2+^]_i_ and enhances BAT activity may through PKA phosphorylation-mediated UCP1 expression in sWAT. It is still not clear that how TRPM8 activation-mediated [Ca^2+^]_i_ increases phosphorylate PKA. Further study is warranted to clarify TRPM8-mediated calcium signaling in adipocytes.

Our present study suggested that dietary menthol leads to an elevation of the thermogenic gene program in cultured white adipocytes and sWAT, which could provide therapeutic benefits for obesity and metabolic diseases. Moreover, *in vivo* data revealed that dietary menthol prevented HFD-induced obesity and ameliorated insulin resistance. Previous study has reported that TRPM8 is widely expressed in many tissues, such as BAT [28], dorsal root ganglia neurons [45], and bladder [46]. Therefore, we could not exclude the contribution of other tissues in the effects of dietary menthol on HFD-induced obesity and insulin resistance. On the other hand, the size of the therapeutic window of menthol in metabolic diseases may depend on the expression of TRPM8 in those tissues.

In conclusion, our study clearly established a novel role for TRPM8 in the thermogenic function of WAT. With the recent reports that TRPM8 is expressed in human WAT [30], menthol-induced TRPM8 activation could mimic cold stimulation-induced thermogenesis and could constitute an intriguing approach to treat human obesity and related metabolic disorders.

## MATERIALS AND METHODS

### Animals

Male C57Bl/6 mice (Shanghai Research Center for Model Organisms, Shanghai, China) were housed in a controlled environment (12 h light/dark cycle; 22-24°C; 50-60% humidity) with food and water *ad libitum*. For all *in vivo* experiments, wild-type (WT) male mice were individually housed in controlled environment. Mice received a normal diet (ND), HFD or HFD plus 1% menthol (HFD+menthol) for continuous 12 weeks from 6-week-old age. All animal protocols were approved by the Animal Research Committee of Shenzhen University (Shenzhen, China), and were performed in accordance with institutional guidelines. Intraperitoneal glucose tolerance tests (IPGTT) were performed by injection of glucose (2 g/kg) after HFD treatment. Insulin tolerance tests (ITT) were conducted after HFD treatment. Porcine insulin (1 unit/kg body weight) was injected into the intraperitoneal space. Blood glucose levels were measured before and 15, 30, 45, 60 and 120 min after glucose or insulin administration.

### Primary culture of white adipocytes from subcutaneous WAT

Primary culture of mouse white adipocytes was carried out according to previously reported methods with slight modification [38]. In brief, pre-adipocytes were isolated from sWAT of three male mice (6-week-old). After getting confluent, pre-adipocytes were inducted in standard medium supplemented with 500 μM 3-isobutyl-1-methylxanthine, 1 μM dexamethasone and 1 μg/ml insulin (induction medium) at the standard incubation condition for 2 days, and then cells were differentiated in standard medium supplemented with 1 μg/ml insulin (differentiation medium) for 6 more days. For all the pharmacological studies, compounds were applied in differentiation medium for 4 h after 8 days of differentiation.

### RT-PCR

Total RNA was isolated using Trizol reagent (Invitrogen, CA, USA) according to the manufacturer’s protocol. In brief, mouse tissues or cells were freshly collected and homogenized in Trizol reagent on ice. RT-PCR was performed using the SuperScript® III kit (Invitrogen, Carlsbad, CA, USA). The RNA was digested with RNase H at 37°C for 20 min. The primer sequence information was reported previously [24].

### Quantitative real-time RT-PCR

Copy numbers of mouse genes were determined by quantitative RT-PCR (qRT-PCR) using SYBR Green MASTER (Invitrogen, Carlsbad, CA, USA) following the manufacturer’s protocol. Data were collected during each extension phase of the PCR reaction and analyzed using ABI-7500 software (Applied Biosystems, CA, USA). The results were standardized for comparison by measuring levels of *36B4* mRNA in each sample. The primer sequence information was reported previously [24].

### Western blotting

White adipocytes were collected and lysed in 100 μL RIPA lysis buffer with complete protease inhibitor cocktail (Roche Molecular Biochemicals, Basel, Switzerland). HEK293 cells transfected with *mTrpm8* plasmid DNA were used for the positive control. Cells were lysed in 100 μL RIPA lysis buffer with protease inhibitor cocktail. The supernatants were collected after centrifugation. The samples were denaturized at 95°C for 5 min and separated on an 8% SDS-PAGE gel and transferred onto a PVDF membrane. The membrane was blocked using BSA reagent (Sigma, St. Louis, USA) at 4°C overnight and then incubated with each antibody at room temperature for 1 h. After three washes with PBS-T (0.1% Triton X-100), the membrane was incubated at room temperature for 1 h with an anti-rabbit IgG or anti-mouse IgG HRP-linked antibody (Cell Signaling Technology, Boston, MA, USA) diluted 1:5000. The signals were visualized with an ECL kit (Pierce, IL, USA) and the PVDF membrane was photographed using a ChemiDoc™ XRS+ imaging system (Bio-rad, California, USA).

### Immunohistochemistry

sWAT isolated from mice treated with ND, HFD and HFD+menthol was fixed in 4% paraformaldehyde/PBS, embedded in paraffin wax and sectioned at 5 μm. sWAT sections were then de-paraffinized in xylene. Briefly, the sections were deparaffinized in xylene and rehydrated through graded ethanol. After microwave antigen retrieval, immunoreactivity was detected by streptavidin-biotin-peroxidase complex method (Fuzhou Maixin, Fuzhou, China) with color development using 3, 3’-diaminobenzidine. The primary antibody was mouse anti-UCP1 (Sigma, St. Louis, USA). Sections were counterstained with Mayer’s hematoxylin for 30 s. Sections of normal hepatic tissue were used as a positive control. The sections were finally penetrated with xylene for 5 min, 3 times, mounted and observed by light microscopy (Olympus, Tokyo, Japan). Adipocyte sizes were assessed by measuring the diameter of adipocyte sizes using Image J software (National Institutes of Health, Bethesda, USA).

### Ca^2+^-imaging

[Ca^2+^]_i_ were monitored by loading primary cultured white adipocytes with Fura-2 AM fluorescent dye (Invitrogen, Carlsbad, CA, USA). Adipocytes were incubated with 5 μM Fura-2 AM for 30 min and used in experiments within 3 h. Fluorescent signals were collected with a CCD camera (Hamamatsu Photonics, Hamamatsu, Japan) and recorded by IP Lab software (Scanalytics, Inc., Rockville, MD, USA) at three sec intervals. The bath solution contained 140 mM NaCl, 5 mM KCl, 2 mM MgCl_2_, 2 mM CaCl_2_, 10 mM HEPES and 10 mM glucose, pH = 7.4, adjusted with NaOH. Cell viability was confirmed with 5 μM ionomycin. All the experiments were performed at room temperature.

### Blood biochemical measurements

Blood glucose levels were measured using a glucose monitor (ACCU-CHEK, Shanghai, China), and plasma insulin levels were examined using ELISA kit (Sigma, St. Louis, USA). Total cholesterol and non-esterified fatty acid (NEFA) levels were measured using commercial kits following instructions from the manufacturer (Sigma, St. Louis, USA).

### Statistical analysis

Group data are presented as the mean ± SEM. Statistical analysis was performed with Student’s *t*-tests or one-way ANOVA followed by multiple *t*-tests with Bonferroni correction using Origin 8.5 software. Only two-tailed *P* values less than 0.05 were considered to represent a significant difference.

## SUPPLEMENTARY MATERIALS FIGURES


