# Supplementary material for: Dietary menthol-induced TRPM8 activation enhances WAT “browning” and ameliorates diet-induced obesity
Source: Oncotarget. 2017 Aug 24;8(43):75114–26. doi: 10.18632/oncotarget.20540 (PMC5650405; doi:10.18632/oncotarget.20540)
Supplement: Supplementary file 1 [file oncotarget-08-75114-s001.pdf]

Dietary menthol-induced TRPM8 activation enhances WAT "browning" and ameliorates diet-induced obesity

SUPPLEMENTARY MATERIALS

Oil red O staining and triglyceride level measurement of brown adipocytes

Oil red O staining was performed using oil red O dye (Sigma, St. Louis, USA). In brief, differentiated white adipocytes were fixed with 4% formalin and incubated at room temperature for at least 1 hr. After fixation, cells were washed with purified water twice and washed with 60% isopropanol at room temperature for 5 min. The cells were let dried completely at room temperature, and oil red O solution was added and then incubated at room temperature for 10 min. Oil red O solution was removed with addition of purified water immediately, and the cells were washed 4 times with purified water. Images were acquired under the microscope (Olympus, Tokyo, Japan) for analysis. For the measurement of triglyceride levels, all the water was removed and cells were dried completely. Oil red O dye was eluted with 100% isopropanol and incubated with gently shaking for 10 min. The OD values were measured at 490 nm using a multi-scan spectrum (Thermo Scientific, Waltham, MA, USA) with 100% isopropanol as a blank.

The number of differentiated adipocytes counting

Differentiated white adipocytes were counted in the pictures taken under the microscope. At least six randomly fields were chosen in each dish. Adipocytes could be distinguished from pre-adipocytes by the presence of visible lipid droplets. For better visualization, lipids were stained with oil red O and only cells positive for this stain were considered as differentiated adipocytes.

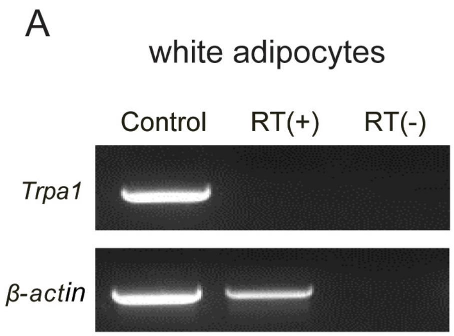

Supplementary Figure 1: mRNA expression of *Trpa1* in adipocytes from mice. (A) RT–PCR analysis of the expression of *β-actin* and *Trpa1* using mouse differentiated white adipocytes with (RT (+)) and without (RT (-)) reverse transcription (RT). Control (Ct.) lanes indicate the results with each plasmid DNA as a template.

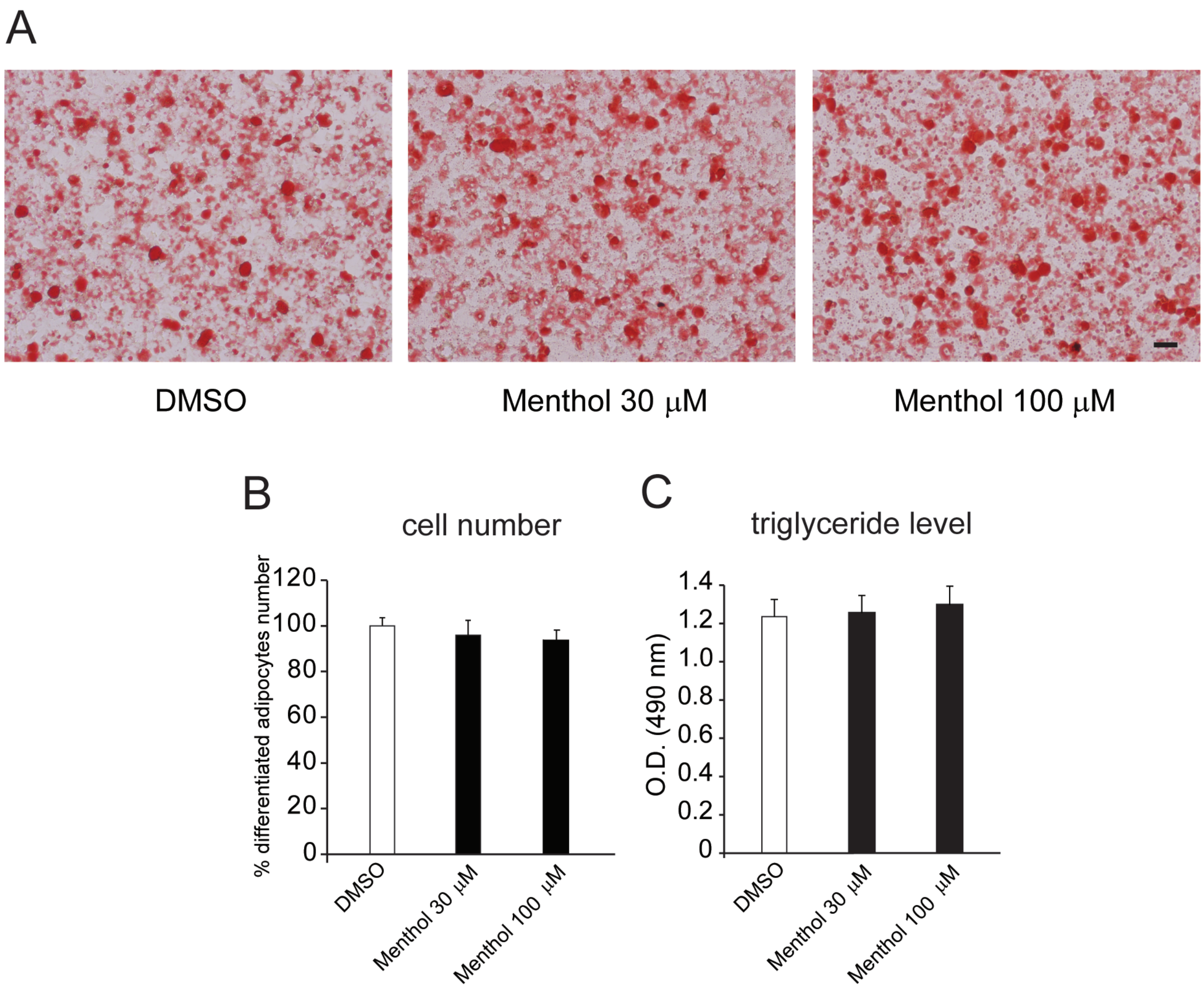

Supplementary Figure 2: Effects of menthol on white adipocyte differentiation. **(A)** Oil red O staining of differentiated mouse white adipocytes treated with different concentrations of menthol. **(B)** and **(C)** Comparison of the numbers of differentiated white adipocytes (B) and triglyceride levels (C) in the cells treated with different concentrations of menthol. Mean ± SEM, n = 6. One-way ANOVA followed by 2-tailed *t*-test with Bonferroni correction.
